# Notes on Formic Aldehyde

**Published:** 1896-05

**Authors:** J. Morgan Howe

**Affiliations:** New York


### NOTES ON FORMIC ALDEHYDE.[1]

BY J. MORGAN HOWE, M.D., M.D.S., NEW YORK.

Having been appointed by the president of this Institute a member of the Committee on Materia Medica and Therapeutics, these notes on this comparatively new therapeutic and prophylactic agent will be presented as a partial report from that committee, with the understanding that the other members will make additions to it during the discussion of the subject.

---

[1] Read before The New York Institute of Stomatology, March 3, 1896.

As this substance has already been shown to have very valuable properties for our service, it is well worthy of our study. The results of my own use of it during the past year have been so favorable that I have looked somewhat into its chemical characteristics, and its literature. These have increased my interest in it, and have helped establish the conviction that it is destined to be of great service to us in several ways.

Formic aldehyde, or formaldehyde, is a gas, prepared by subjecting methyl alcohol (or wood alcohol) to oxidation. The chemical formula of methyl alcohol is $CH_4O$, of formaldehyde is $CH_2O$, and of formic acid is $CH_2O_2$. It is a product midway in the process of oxidation, between the alcohol and the acid. The first step in the oxygenizing process is effected by the loss of two atoms of hydrogen, and this change is expressed in the word aldehyde, contracted from *alcohol dehydrogenatum.* The next step in the oxygenizing process, which forms the acid, is effected by the addition of an atom of oxygen, as is seen by the formula.

As this gas is readily absorbed by water it is furnished to us in a forty-per-cent. solution, and in this condition is known also as formalin and as formol. As formalin is quite commonly used as a designation, and is, perhaps, a trifle more convenient, it is not objectionable, excepting its tendency to cause the chemical constitution of the compound to be lost sight of; for aldehydes, being as regular chemical compounds as alcohols or acids, are entitled, as such, to have their regular designations remembered. For this reason I have chosen to use the chemical name at the head of these notes.

Formaldehyde in solution, as we obtain it commercially, is comparable in a way to the solution of ammonia, with which we are all so familiar,—a gas absorbed by water, and capable of being diluted with water to any extent, so that any desired percentage may be obtained in that way.

Although its antibacterial action was noticed by Löw in 1886, it has only begun to attract much attention during the last two or three years. Berlioz and Trillat have demonstrated that anthrax bacilli were killed by a dilution of 1 to 50,000, and Drs. Slater and Rideal have shown that the growth of different specific micro-organisms was inhibited by its addition to the media in the proportions of 1 to 5000 up to 1 to 20,000; and soiled cloths from the post-mortem room were rendered sterile by soaking in a one-per-cent. solution.

A one-half-per-cent. solution has been used in surgical operations for irrigating wounds and washing the hands and instruments of the operators, with better results than when other antiseptics were used for those purposes, and it has been used in a similar way in ophthalmic practice. Dr. J. M. Davidson, of England, has recently reported very favorable results from the use of solutions of 1 to 2000 and 1 to 3000 in abrasions of the cornea, and in hypopyon ulcers. It prevents suppuration and relieves pain, producing no irritation in the strength recommended, according to his experience. Whooping-cough has been successfully treated by spraying a one-per-cent. solution over the heads of the patients; and experiments are being made similarly in the treatment of pulmonary consumption.

In bacteriology formaldehyde has been found of great service in the arrest and fixation of development, at any desired stage; and for preserving and hardening anatomical and microscopical specimens it has served the purpose better than either alcohol or Müller's fluid.

This agent has been proved to have such extraordinary disinfecting power, together with non-toxic qualities, that one-half-per-cent. solutions are recommended as fully adequate for the general disinfection of sick-rooms, infected clothing, books, and furniture, by means of spraying apparatus; and for the disinfection and deodorization of vessels, closets, waste-pipes, etc., and their contents, by simply pouring over them. A one-per-cent. solution has been found amply sufficient for almost all disinfecting requirements, and from one-fourth- to three-fourths-per-cent. solutions are strong enough for most purposes. In these latter dilutions Professor Cohn has found it capable of preventing the development of the bacteria of putrefaction, and aqueous infusions thick with bacterial life were cleared by such additions, the dead bacteria sinking gradually to the bottom, and the offensive odor disappearing.

Formaldehyde has been used to some extent as a preservative of milk in Great Britain. It has four times the potency of any other antiseptic commonly used for that purpose. Four or five drops of the forty-per-cent. solution has prevented the souring of one hundred cubic centimetres of milk for six weeks. If it has been used for such purposes in this country, the fact does not appear to be generally known, for it was not mentioned by physicians discussing the adulteration of milk, at a meeting specially considering that subject, held last January, in the New York Academy of Medicine.

My attention was first called to formaldehyde, or formalin, by the remarks made by Dr. Louis Jack at a meeting of the Academy of Stomatology, and reported in the INTERNATIONAL DENTAL JOUR-

NAL for March, 1895; in which he related the cure of a pulpless, irritable tooth, that would not bear stopping up, by means of two applications of this remedy. It appears that Dr. Kirk had spoken of this substance to Dr. Jack. He having learned of it from Dr. Mascort, who had treated indiscriminately many cases of alveolar abscess with it, at the St. Louis Hospital, and was quite enthusiastic over its use "as a root-dressing for putrescent cases."

On March 28, 1895, Miss B. (an invalid) called for the treatment of a lower bicuspid that was pulpless, and in that peculiar, irritable condition that will not endure the occlusion of the pulp-canal. The family had been my patients for some years, but this member had been referred to another dentist nearer to her home, because her poor health prevented her from being certain of ability to keep appointments, and this particular tooth had been—at the time of her call—under treatment for three months. The tooth was treated for several days before I recalled the reference to formalin I had read nearly a month before, and during this time various remedies were used with some apparent benefit at first, but later with renewed increase of irritability.

At this time, when convinced that I had gained nothing by what I had done, I looked up the record and obtained some formalin in five-per-cent. solution. The first application was made on cotton in the root-canal, and covered with cotton saturated with sandarach, so that the patient could remove it if necessary. She caught cold next day, and sent word she could not keep her engagement, and I did not see her for nine days. When she called again the dressing was *in situ* as I had placed it, and she had felt no discomfort from it. I renewed the dressing, covering it this time with a temporary gutta-percha stopping, and six days later the root was filled with oxychloride of zinc. This tooth gave no trouble after the first dressing of formalin was placed in the root-canal, and has continued perfectly comfortable and useful until the present time.

I have since had several similar cases to treat, and the application of formalin in five-per-cent. solution has been successful in all but one. The key-note of the remarkable and unique influence of this remedy is struck more clearly, I think, in this particular class of cases than in any other. No other medicament that I know of has such an effect. In the case described at length above, the other dressings used were impotent to enable the tooth to bear occlusion of the vent for more than a few hours at the best.

The exceptional case referred to was a cuspid that had been open and discharging slightly for several years, and careful examination convinced me that there was an opening through the side of the root near the apex.

Since using formalin successfully in the first one or two peculiar cases already referred to, I have gradually used it more indiscriminately in the treatment of pulpless teeth, without fistulæ, and have found it peculiarly and wonderfully effective in disinfecting them, and removing all tendency to irritation from admission of air, or efforts to cleanse and prepare the root-canals, or from subsequent filling of the roots.

After considerable use of this substance, I discovered that it was possessed of decided deodorizing power, before I had consulted its literature sufficiently to find that this quality is claimed for it, as has been noted above. In one recent case in which I tested it, an offensive molar was treated in the root-canals with electrozone, without removing the bad odor, but a two-and-a-half-per-cent. solution of formalin made it quite free from all offensiveness in a few minutes.

I have not used it in the treatment of alveolar abscesses with fistulæ, but have tested its efficacy a few times in pyorrhœa alveolaris, and found that it seemed to have no special influence to diminish the formation of pus. My judgment of its therapeutic influence on tissues, to which it is applied, is that its special sphere is to allay irritability of the tissue and prevent inflammation and suppuration. It may have greater influence in reducing suppuration, and in favoring a return to normal conditions after pus has formed than I have discovered, and I hope that something more definite on this point will be reported in the discussion of this subject.

It is a powerful irritant, and the warning that Dr. Jack gave in the record before referred to—that is, that it should not be used in greater strength than a five-per-cent. solution—may well be heeded. Its application in root-canals in that dilution will sometimes cause pain, and I have even found it to give evidence of irritation in a two-and-a-half-per-cent. solution. But the pain has always passed away in an hour or two, when the dressing was left in the tooth, and has ceased in a few moments after the removal of the dressing, in those cases that caused complaint at the time. These evidences of irritation have been only in exceptional cases, and I have concluded, from my clinical experience, that it is most likely to cause pain when it is applied in the root-canals of teeth whose pulps have been recently destroyed; for which other dressings would have been better. The inference is that its irritating properties are overcome by chemical action when it is brought into contact with putrescent matter. In my recent use of formalin I have found a two-and-a-half-per-cent. solution answer the purpose so well, both as a deodorizer and disinfectant, that I am inclined to believe that a stronger application is unnecessary. I have disinfected many putrescent pulp-canals with this substance in the two-and-a-half-per-cent. as well as the five-per-cent. solution, and filled the roots at subsequent sittings without the use of any other agent whatever for treatment, and have had remarkably satisfactory results.

In two cases only pain and swelling to a very limited degree followed the filling of the roots, and this I attributed to my own or the patient's anxiety to get the work finished, and off the list; being due rather to an error of judgment in filling too soon than to lack of efficacy in the formalin dressing. Formalin has some coagulating action on albumen, which is apparent even in the action of the two-and-a-half-per-cent. solution a few minutes after its contact with the white of egg. But the coagulating action of the forty-per-cent. solution is very much less decided than pure carbolic acid. Electrozone has no coagulating effect whatever. A five-per-cent. formalin solution added to meat broth,—that had been infected by a broach used in a putrescent pulp-canal,—and allowed to become putrid, showed in a test-tube much more vigor of chemical action and rapidity of clearing of the infection than did a similar test of electrozone added to the same putrescent liquid. I have found formalin to be very efficacious in disinfecting, without causing irritation, those cases of quiescent pulpless teeth whose pulp-cavities demand opening, but are very liable to have pericementitis supervene as a result of such opening.

When I was a young man, I was very much distressed by the evidence that was often presented to me that I had precipitated pain and suffering upon my confiding patients by opening their pulpless teeth. But I found, to my astonishment, that those whom I considered wise men could give me no special light or encouragement on the subject. Indeed, most of them denied that the opening of such teeth was the cause of any special solicitude to them. They applied some medicament which in their hands always prevented trouble, and each one had a different remedy; but in my hands afterwards none of these agents proved of sure value in preventing inflammation, as the result of opening pulp-canals with putrid contents, that had been closed and were not causing irritation at the time.

Dr. James Truman wrote in the "American System of Dentistry," 1885, "A dead pulp may remain for years—and it may be for life—very quiet if not exposed by caries, but may, in a few hours, produce violent pericementitis if exposed to the action of the atmosphere. It is, therefore, oftentimes a question in the diagnosis of such a tooth whether the great risk warrants meddling with it at all."

I think there is now a general recognition of the danger attending this procedure, but I fear it is not by any means universal among dentists, and I call attention to it to emphasize the importance of its being recognized, as well as to record the usefulness of formalin as an efficient preventive.

I had not found anything to serve the purpose as well as iodoform dissolved in alcohol and ether until the advent of electrozone. In 1884 I read a short paper on antiseptics, in which I referred to iodoform as the most effective safeguard against inflammation, as a result of opening quiescent pulpless teeth, and until I began to use electrozone as a disinfectant I had not dared to rely upon anything else in such cases. I have used the latter, however, with satisfaction ever since its introduction, and it undoubtedly has a certain soothing and reducing influence on inflamed tissues, besides its antiseptic power.

In exactly what cases, and in what conditions, solutions of formaldehyde will serve better than electrozone or other agents it will take some time to determine. Each remedial agent that we have has its limitations, and each has a sphere of influence that makes it better adapted to certain conditions than anything else would be, but there is no doubt that formaldehyde is a valuable addition to our list of medicaments, and it is probably destined to take the place of most of the prophylactics we have used heretofore. It simplifies and renders easy the sterilization of instruments and of everything that needs disinfection, and there can be no doubt that a one-half-per-cent solution is perfectly efficient for such purposes after the article has been previously cleansed in an ordinary way. Such a solution does no harm to steel instruments, other than results from the application of water, and Drs. De Buck and Vanderlinden, of Ghent, have found it more efficient than a two-and-a-half-per-cent. carbolic or a 1 to 500 sublimate solution.

I have noticed that an antiseptic lotion advertised for a mouth-wash has in the published list of ingredients a 1 to 1000 solution of formaldehyde in a proportion not given. I find that a one-half-per-cent. solution of formaldehyde, if rinsed vigorously about the mouth, produces a stinging sensation, suggesting the effects of capsicum, and even a one-fourth-per-cent. solution does not cease to have a similar effect on the mouth and fauces, although the sensation of astringent action is also perceptible with the latter. As far as antiseptic power is concerned, we may certainly count upon its value here also, but what medicinal effect it will have on the mucous membranes and gum tissues will have to be determined by experimental use.

It is apparent that this substance has a medicinal influence aside from its antiseptic effects, and that a great portion of its value to us depends upon its peculiar medicinal action. The irritable condition of a pulpless tooth that will not bear stopping up is not amenable to control by antiseptic treatment alone, unless the agent used has also a medicinal influence on the tissues. And the same thing is true regarding the contents of pulp-canals in pulpless teeth that threaten to produce irritation by access of air or germs. The application of the most potent antiseptic is not so certain to avert unfavorable symptoms as is the use of iodoform, which is rather low down in the antiseptic scale, but which has a certain power aside from germicidal action that produces a beneficial effect on living tissue. In other words, the usefulness of medicaments, suitable for the treatment of abnormal conditions of living tissue, cannot be expressed in terms of antiseptic power. The latter has engrossed attention to such a degree during recent years as almost to exclude the fact altogether that almost every agent used has a medicinal or toxic action, which may be beneficial or the reverse.

The preserving and hardening effects on tissues that have been manifested by formaldehyde suggest that it may be found of service in efforts to preserve and make innocuous those dead pulps, or portions of pulps, that circumstances make it desirable or expedient to leave *in situ*. Any simple and quick means of accomplishing this would result in such great saving, to both patient and dentist, that all must regard it as a desideratum.

Probably we would all agree in the opinion that no treatment of a pulpless tooth can ever fulfil the requirements of placing it in the safest condition for the longest time, as does the complete filling of the root-canals with an impermeable substance; but there is a possibility of approximation to the ideal condition of safety by some other less arduous and expensive method. Several experimenters and writers have discussed this subject in recent years, and I hope this new agent—formaldehyde—may serve to forward to success efforts in this direction. The teeth of the multitude, who cannot afford either time or money for difficult and expensive operations, need to be saved by simple and inexpensive methods, and I am sure we all sympathize with every effort to render the process of saving teeth easy and simple.

In commending formaldehyde to your attention and study, it is with the conviction that it is to be a very valuable addition to our equipment of therapeutic agents. The limitations of its usefulness will soon be discovered, but at present its value is undoubted in the treatment of foul pulp-canals, and irritable apical tissues, and in the disinfection of anything that needs such cleansing.

